# Triazolo pyrimidine derivatives of coumarin and benzocoumarin: green synthesis, biological activity screening study with in silico evaluation

**DOI:** 10.1186/s13065-025-01636-6

**Published:** 2025-10-14

**Authors:** Hawazen M. Hassanain, Meaad J. Al-Zahrani, Roaa M. Alreemi, Huda A. Al-Ghamdi, Ahlam I. Al-Sulami, Khadijah M. Al-Zaydi

**Affiliations:** 1 Department of Chemistry, College of Science, University of Jeddah, P.O. Box 80327, Jeddah, 21589 Saudi Arabia; 2 Department of Biological Sciences, College of Science, University of Jeddah, P.O. Box 80327, Jeddah, 21589 Saudi Arabia

**Keywords:** Triazolo pyrimidine, Coumarin, Benzo-coumarin, In silico, Coronavirus, ADME

## Abstract

In the current study, triazolo pyrimidine coumarin and benzocoumarin derivatives were prepared using green methods as a fast and cost-efficient strategy in comparison with the traditional method in terms of reaction time and yield. Combining coumarins with other bioactive compounds could improve the anticancer properties and other biological activities. Therefore, both compounds were evaluated in silico as potential early-stage small molecule inhibitors of three targets, which cause four life-threatening diseases: leukaemia FLT3 (PDB: 4XUF), SARS-COV-2 (3CLpro) (PDB: 6M2N), and adenosine A1 receptors A1R (PDB: 5N2S), which could cause heart failure and Alzheimer’s disease. It was discovered that both compounds show promising results against the three targets. However, benzocoumarin shows an advantage over coumarin compounds. The effects of modifications to the coumarin core have been studied using an in silico technique that evaluates pharmacokinetics, absorption, distribution, metabolism, excretion, and toxicity of these compounds, showing promising results as an early-stage drug. However, both compounds were predicted to be highly absorbed from the gastrointestinal tract and enter the blood-brain barrier efficiently. The predicted oral toxicity of both compounds was LD50 = 350 mg/kg, putting both of them in the IV toxicity class. The optical properties of coumarin and benzocoumarin compounds were also investigated.

**Graphical abstract:**

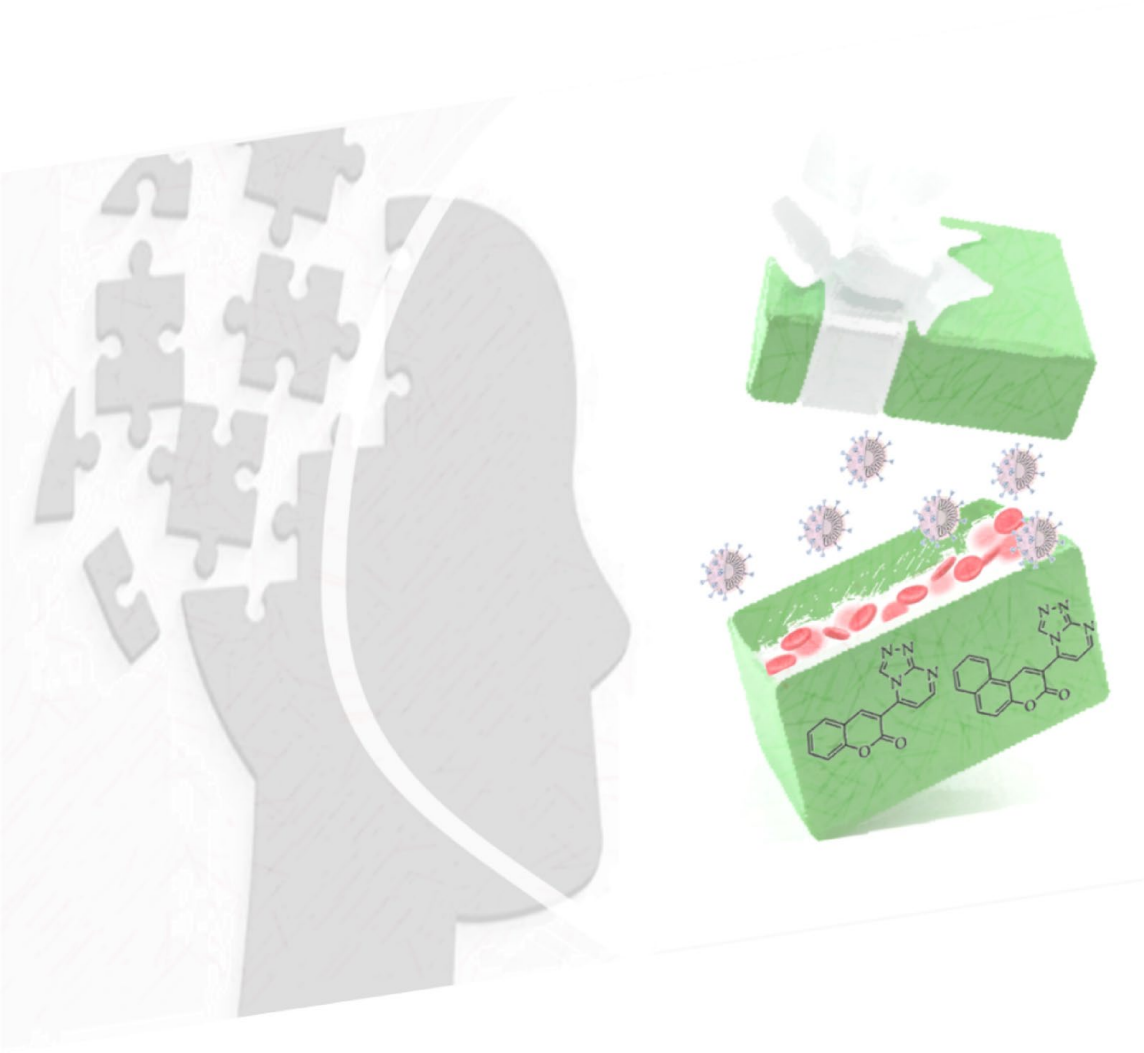

**Supplementary Information:**

The online version contains supplementary material available at 10.1186/s13065-025-01636-6.

## Introduction

Coumarins are a class of heterocyclic compounds that have been intensively studied due to their applications in biology and pharmaceutical chemistry [1–3]. Coumarin compounds are commonly found in cosmetics, perfumes, pharmaceuticals, and food additives due to their extensive antibacterial, anthelmintic, antioxidant, anticonvulsant, anti-cancer, anti-inflammatory, anti-HIV, and antineoplastic effects, as well as their interesting structural characteristics [1–3]. Furthermore, coumarins play an important role in fluorescent biomolecule labelling, metal ion detection, microenvironment polarity detection, pH measurement, laser dyes, and optical brighteners [4–6]. Therefore, modifying the coumarin core is a common strategy to create new coumarin compounds with specific desired properties, particularly for applications in fluorescent probes, pharmaceuticals, and materials science [7].

It should be noted that coumarins and their derivatives have received a lot of attention for their anti-tumour potential due to their cytotoxic action against a wide range of cancer cell types, including gastric, liver, colon, breast, prostate, periodontal, fibroblast, nasopharyngeal, and normal fibroblast cell lines [8–10]. Furthermore, coumarin derivatives can suppress growth in human cancer cell lines, including kidney (ACHN), lung (A549, H727), leukaemia (HL-60), breast (MCF7), and renal cell carcinoma [8–10]. The addition of various substituents can have a substantial impact on the biological and pharmacological [7], optical and solid-state behaviour of coumarins [4–6].

Benzocoumarins are coumarins with a phenyl group bonded to 3,4-, 5,6-, 6,7-, or 7,8-positions. They are can be categorized into four types depending on the position of the fused aromatic ring in the parent coumarin backbone: (1) benzo[c]coumarin (3,4-benzocoumarin) ; (2) benzo[g]coumarin (6,7-benzocoumarin); (3) benzo[f]coumarin (5,6-benzocoumarin); and (4) benzo[h]coumarin (7,8-benzocoumarin) [11, 12]. Benzocoumarin exhibits antimicrobial and anti-inflammatory activity [15, 16]. Also, benzocoumarin could function as an antagonist of cannabinoid receptors and an inhibitor of monoamine oxidase [14]. Moreover, combining coumarins with other bioactive compounds, such as pyrazole and pyrazolo[1,5a]pyrimidine, triazole, has recently been demonstrated to possess considerable anticancer properties [8, 13, 14].

On the other hand, 1,2,4-Triazoles and pyrimidines are significant heterocyclic compounds recognised for their diverse biological activities, which make them valuable in medicinal chemistry and drug discovery. 1,2,4-Triazoles are particularly noted for their antifungal, antibacterial, anticancer, anticonvulsant, and antiviral properties [15–19]. Similarly, pyrimidines serve as essential components of nucleic acids and as bioactive molecules, demonstrating a broad range of biological activities, including anti-inflammatory, anti-malarial, anti-tumour, and antiviral effects [15, 20, 21].

**Scheme 1:**
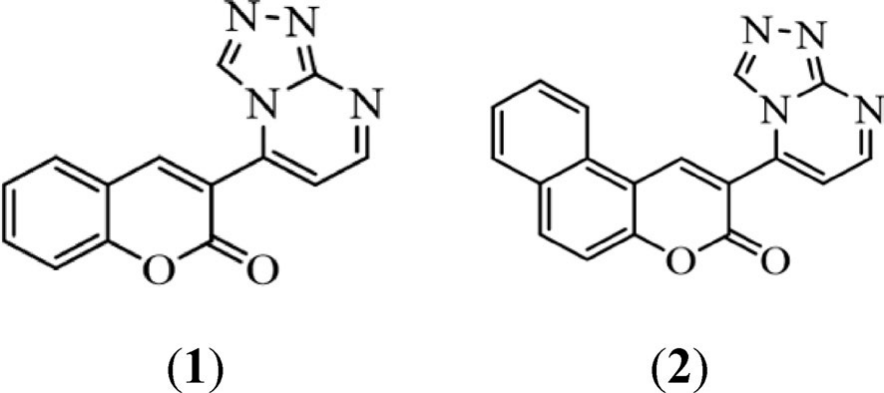
Coumarin derivatives used in this study

In this paper, we have focused on two types of coumarin derivatives, namely triazolo pyrimidine coumarin and triazolo pyrimidine benzo[f]coumarin (benzocoumarin), as shown in Scheme 1. Four life-threatening diseases were studied: Leukemia is a complicated type of blood malignancy that is defined by an uncontrolled proliferation of aberrant blood cells. Acute myeloid leukemia (AML) is one of the most aggressive and difficult-to-treat types of leukemia in which abnormal blood cells multiply without control [24, 25]. The receptor tyrosine kinase, which is encoded by the FLT3 gene, is essential for healthy hematopoiesis. FLT3 gene mutations result in aberrant CELL signaling leading to increased proliferation, reduced apoptosis, and bone marrow accumulation of malignant cells [26, 27]. Therefore, FLT3 is an important target of therapeutic intervention and developing targeted inhibitors of FLT3 to improve outcomes in AML patients. It should be noted that coumarin has shown positive results to reduce the negative effects of radiation therapy and is commonly used to treat leukemia, prostate cancer, and renal cell carcinoma [7]. However, according to our knowledge, no study showed the effect of coumarin compounds as FLT3 targets. The current study intends to investigate the efficacy of new coumarin triazolo pyrimidine derivatives as anticancer medicines by specifically targeting FLT3 and disrupting the related cell signalling of carcinogenesis using an in silico approach.

The World Health Organisation (WHO) has classified COVID-19, caused by the SARS-CoV-2 virus, as a pandemic due to its contagious and potentially fatal nature [28, 29]. This infectious disease can lead to a wide range of respiratory issues, from mild symptoms to severe pneumonia, particularly affecting older adults and those with underlying health conditions [40].The outbreak has spurred significant research efforts for vaccines and antiviral treatments, impacting not just public health but the global economy as well [30, 31]. Key proteins involved in the virus’s replication process include the proteases 3CLpro and PLpro, which are crucial for processing the viral RNA genome’s polyproteins [32, 33]. Particularly, 3CLpro is a cysteine protease that cleaves these polyproteins [34–36]. SARS-CoV-2 3CL protease (3CLpro) is an important target for many therapeutic agents of COVED-19. This enzyme is important in the virus life cycle through its role in the conversion of viral polyproteins into functional proteins essential for viral replication [37, 38]. As a result, it is considered a promising target for the development of antiviral therapies [35, 39–41]. Additionally, there is no recognized human homologue protein with a cleavage site similar to 3CLpro, reducing the risk of off-target effects and making the development of 3CLpro inhibitors an interesting COVID-19 therapy option [34, 42].

Adenosine A1 receptors (A1R) are G-protein-coupled receptors involved in the regulation of several physiological processes, such as neurotransmission modulation, inflammatory control, and cardiac protection [43, 44]. Due to their participation in numerous pathological processes, A1Rs have become a promising target for therapeutic interventions. For example, A1R inhibition has shown potential cardioprotective effects in the case of heart failure [45]. Furthermore, in neurological disorders like Alzheimer’s, inhibition of A1R stimulates the release of neurotransmitters, enhances cognitive function, and might help decrease symptoms associated with neurodegeneration [46]. Targeting Adenosine A1 receptors by the two coumarin triazolo pyrimidine derivatives via interaction with the binding centre of A1R, according to in silico investigation, provides a therapeutic avenue for heart failure and Alzheimer’s and other disorders related to A1R inhibition. It should be noted that several studies focus on the inhibitory activity of coumarin compounds against A1 receptors [47, 48].

In this paper, we have focused on two types of coumarin derivatives, namely triazolo pyrimidine coumarin and triazolo pyrimidine benzo[f]coumarin (benzocoumarin), as shown in Scheme 1. Then, we will evaluate the photophysical properties and biological activity of such compounds. pharmacological and absorption, distribution, metabolism, excretion, and toxicological properties of structures will be investigated (ADMET). The in-silico approach was chosen in this study because the development of a new drug or vaccine is often time-consuming, as it must be thoroughly evaluated and proven safe in clinical trials before being approved for human use [22, 23]. Additionally, we evaluate our compounds in silico against three targes that cause four life-threatening diseases: leukaemia, SARS-CoV-2 3CL protease, and adenosine A1 receptors, which could cause heart failure and Alzheimer’s disease.

## Materials and methods

### General experimental

Reagent grade chemicals were acquired from Sigma-Aldrich and Fisher Scientific UK, and used without additional purification. Melting points were determined with a Gallenkamp electrothermal apparatus. NMR spectra (^1^H and ^13^C) were recorded at room temperature in DMSO on 850 MHz and 600 MHz spectrometers, with chemical shifts reported in δ relative to the solvent. Infrared spectroscopy was performed using a Spectrum Two FT-IR spectrometer, covering 8300 to 350 cm⁻¹. Ultrasound irradiation utilised a 2004 high-intensity ultrasonic processor with a 750 W temperature controller and 25 kHz frequency. Reaction temperatures were manually set based on the solvent’s boiling point. Q-Tube-assisted reactions were conducted in a Q-Tube-safe reactor from Q Labtech. Monitoring of reactions was performed using TLC silica gel plates from Sigma-Aldrich with a fluorescent indicator at 254 nm, employing a solvent system of ethyl acetate and hexane in a 9:1 or 9.5:0.5 ratio.

### Synthesis

*Synthesis of compounds* ***A*** compound were prepared according to literature Prep [28]. Dimethylformamide dimethylacetal (DMF DMA, 0.12 mol) was added to 3-acetylcoumarin (0.1 mol) in 11 ml of and the mixture was refluxed for 6 h. Solvent were removed under reduced pressure. Then, the solid was collected and recrystallized from ethanol.

*The color* Light orange crystal, m.p.165–166 °C, IR: υ/cm-1 1724 (ester CO), 1664 (ketone CO). ^1^H-NMR (850 MHz, DMSO-d_6_): δ, ppm, 2.87 (s, 3 H, CH3), 3.15 (s, 3 H, CH3), 6.03 (d, 1H), 7.37 (t, 1H, Ar-H), 7.43 (d, 1H-Ar-H), 7.66 (t, 1H, Ar-H), 7.78 (d, 1H), 7.87 (d, Ar-1H), 8.54 (s, 1H, coumarin H-4). ^**13**^**C-NMR** (100 MHz, DMSO-d_6_): δ, ppm, 39.9, 116.4, 117.5, 118.3, 121.9, 122.6, 130.4, 130.8, 145.4, 154.9, 155.4, 159.6, 195.5 .

*Synthesis of compounds* ***B*** compound were prepared according to literature Prep [28]. Dimethylformamide dimethylacetal (DMF DMA, 0.12 mol) was added 2-acetyl benzo[f]coumarin (0.1 mol) in xylene at 100 °C. Then, the mixer were refluxed for 6 h. Removal of the solvent under reduced pressure yielded the crude product which was recrystallized from EtOH/DMF.

*The color* Dark orange crystal. m.p.202–204 °C. IR: υ/cm-1 1724 (ester CO), 1638 (ketone CO). ^1^H-NMR (850 MHz, DMSO-d_6_): δ, ppm, 2.90 (s, 3 H, CH3), 3.17 (s, 3 H, CH3), 6.14 (d, 1H), 7.62 (d, 1H, Ar-H), 7.66 (t, 1H, Ar-H), 7.77 (t, 1H, Ar-H), 7.84 (d, 1H), 8.08 (d, 1H, Ar-H), 8.26 (d, 1H, Ar-H), 8.57 (d, 1H, Ar-H), 9.25(s,1H, benzocoumarin H) .^**13**^**C-NMR** (100 MHz, DMSO-d_6_): δ, ppm, 39.9, 113.8, 117.5, 121.9, 124.1, 126.5, 126.7, 127.5-127.6, 127.5, 127.6, 128.2, 128.3, 133.7, 147.3, 149.4, 155.4, 159.1, 195.5.

General method for synthesis of compound 1, and 2:

*Method I (Conventional heating)* Enaminones coumarin** A** or** B** were added to a mixture of 1*H*-1,2,4-triazol-3-amine, 2-amino-3-cyano-4, 5, 6, 7 tetrahydrobenzo [b]thiophen-5-ylium, 2-aminopyrazin and *p*-toluidine (1 mol each) in (20 ml AcOH/EtOH 1:1), which was refluxed for 5 h to give** 1–2** ,respectively. Then, the solid was collected and recrystallized from ethanol.

*Method II (Q-Tube)* Enaminones coumarin** A**, or** B** (1 mol) was added to a mixture of 1*H*-1,2,4-triazol-3-amine, 2-amino-3-cyano-4, 5, 6, 7 tetrahydrobenzo [b]thiophen-5-ylium, 2-aminopyrazin and *p*-toluidine (1 mol each) in 11 ml (AcOH/EtOH 1:1, at 160 °C ) to give** 1–2** ,respectively. Then, the solid was collected and recrystallized from ethanol.

*Method III (US)* A mixture of enaminones coumarin** A**, or** B**, were added to 1*H*-1,2,4-triazol-3-amine, 2-amino-3-cyano-4, 5, 6, 7 tetrahydrobenzo [b]thiophen-5-ylium, 2-aminopyrazin and p-toluidine (1 mol each) in 50 ml (AcOH/EtOH 1:1, at 90 °C) to give** 1–2** ,respectively. Then, the solid was collected and recrystallized from ethanol.

*Compound 1:The color* pale yellow crystals, Method I (66%), Method II (80%), Method III (99%),** m.p.**250–252 °C,** IR**: υ/cm^-1^ 1724 (ester CO), 1604 (C = N), ^**1**^**H-NMR** (850 MHz, DMSO-*d*_*6*_): δ, ppm, 7.31 (t, 1H, Ar-H), 7.38 (d, 1H, Ar-H), 7.62 (t, 1H, Ar-H), 7.74 (d, 1H, pyrimidine-H) 7.55 (d, 1H, Ar-H), 8.56 (s, 1H, coumarin H-4), 8.75 (s, 1H, triazolo-H), 8.84 (d, 1H, pyrimidine-H). ^**13**^**C-NMR** (150 MHz, DMSO-d_6_): δ, ppm, 39.9, 112.19, 116.96, 117.74, 118, 43, 125.52, 130.39, 134.73, 142.55, 147.60, 154.24, 155.62, 155.87, 157.82. Anal. Calcd. For C14H8N4O2 (264.24): C, 63.64; H, 3.05; N, 21.20; Found : C, 63.74; H, 3.10; N, 21.26.

*Compound 2:The color* dark yellow crystals, Method I (80%), Method II (100%), Method III (96%), m.p.232–233 °C, IR: υ/cm^-1^ 1720 (ester CO), 1607(C = N). ^1^H-NMR (850 MHz, DMSO-*d*_*6*_): δ، ppm, 7.70 (t,1H, Ar-H), 7.74 (d, 1H, Ar-H), 7.83 (t, 1H, Ar-H), 7.85 (d, 1H, pyrimidine-H) 8.15 (d, 1H, Ar-H), 8.40 (d, 1H, Ar-H), 8.54 (d, 1H, Ar-H), 8.79 (s, 1H, triazolo-H), 9.07 (d, 1H, pyrimidine-H), 9.85 (s,1H, benzocoumarin H-4). ^**3**^**C-NMR** (150 MHz, DMSO-d_6_): δ, ppm, 39.9, 112.26, 112.85, 116.55,117.14, 122, 66, 127.14, 129.60, 130.5, 142.84, 143.70, 143.70, 154.78, 155.94, 155.96, 157.83 .Anal. Calcd. For C18H10N4O2 (314.30): C, 68.79; H, 3.21; N, 17.83; Found: C, 68.74; H, 3.10; N, 17.76.

### Docking study

#### Protein preparation

The X-ray structures of targeted proteins were downloaded from the Protein Data Bank (www.rcsb.org). The structure with PDB ID 4XUF corresponds to the Fms-like Tyrosine Kinase 3 (FLT3) [49], PDB ID 6M2N corresponds to SARS-CoV-2 3CL protease (3CL pro) [50], and PDB ID 5N2S corresponds to adenosine A1 receptor (A1R) [51]. The crystal structures of these proteins were prepared with the Protein Preparation Wizard in the Schrodinger suite. The default standard protocol used for preparation includes adding missing hydrogen atoms to residues, correcting metal ionisation states, and removing water molecules > 5 Å from protein residues.

#### Ligand preparation

The two synthetic compounds were prepared for docking using Schrödinger’s LigPrep tool. This tool is utilised to convert the 2D structures of the compounds to 3D structures, energy minimising, hydrogen bonds optimisation and creating all possible ionisation states and tautomeric forms at a pH range of 7.0–2.0. This comprehensive preparation ensures that all potential forms of the ligands are considered during the docking process.

#### Grid generation and molecular docking

The Receptor-Grid-Generation tool from the Schrödinger package was employed to create the grid boxes around the co-crystallised ligands in the crystal structures of the selected proteins. Docking of the two synthetic compounds was carried out within the grid boxes by using the Ligand Docking tool of Schrödinger, employing the extra precision (XP) protocol and all other parameters were left in their default settings. To assess the docking method, re-docking of the co-crystallised ligands was conducted before docking the synthetic compounds.

### Molecular dynamics (MD) simulations

MD simulations were conducted using the Desmond simulation package integrated within the Schrödinger suite. To create an explicit solvent environment, each protein–ligand complex was enclosed in an orthorhombic simulation box filled with TIP3P water molecules, ensuring a minimum distance of 10 Å between the complex and the box boundaries. An appropriate concentration of sodium (Na⁺) and chloride (Cl⁻) ions was incorporated into the system to maintain electrostatic neutrality and approximate physiological ionic strength. The simulation protocol involved a series of preparatory steps, including energy minimisation and system equilibration, following the default relaxation protocols available in Desmond. The production phase of the simulation was executed for 100 nanoseconds (ns) under NPT ensemble conditions to replicate physiological conditions. Temperature was kept at 300 K, while pressure was stabilised at 1.01325 bar. Additionally, the system’s pH was controlled at approximately 7.0 ± 0.2 to reflect a biologically relevant environment.

### Prediction of the pharmacokinetic properties and toxicological properties using ADMET

ADMET play an important role in the drug discovery process since the safety of the drug might cause it to fail. Therefore, investigation of the toxicity of a potential drug in the early stages of development is significant, which prevents the loss of resources and time [52, 53]. Therefore, molecules used in this study were submitted to the ADMET properties. ADMET profile, Physicochemical, pharmacokinetic and drug-likeness evaluation of all compounds (**1**, and **2**) were predicted using Swiss ADME (http://www.swissadme.ch/) [54], and ProTox II (http://www.tox.charite.de/tox) [55, 56]. It should be noted that compound toxicological properties were analysed taking into account the Lipinski, Veber and Pfizer toxicity empirical rules [57].

## Results and discussion

### Synthesis and characterisation

In recent years, different methodologies have been used for the syntheses of coumarin derivatives, including Knoevenagel, Perkin, Wittig, Reformatsky, Pechmann, Kostanecki, Buchwald-Hartwig, and metal-induced coupling reactions, including Heck and Suzuki coupling reactions [6]. Additionally, green approaches involving microwave or ultrasound energy were used for the syntheses of coumarin derivatives [58]. Green methodologies have received a lot of attention in recent years since the economic and environmental benefits of developing cleaner, safer, and more productive synthetic processes [58, 59]. They make synthetic processes clean, safe, and high-yielding. Therefore, the development of environmentally benign organic reactions has become a crucial and demanding research area in modern organic chemical research [59].

The preparation of enaminone **A**, and **B** were prepared according to the literature [60]. Then, a new green methodology was developed for the preparation of 3-([1, 2, 4] [Triazolo[4,3-a]pyrimidin-5-yl)-2 H-chromen-2-one derivative **1**, and 2-([1, 2, 4] Triazolo[4,3-a]pyrimidin-5-yl)-3 H-benzo[f]chromen-3-one derivative **2** by using Q-Tube and US.

**Scheme 2:**
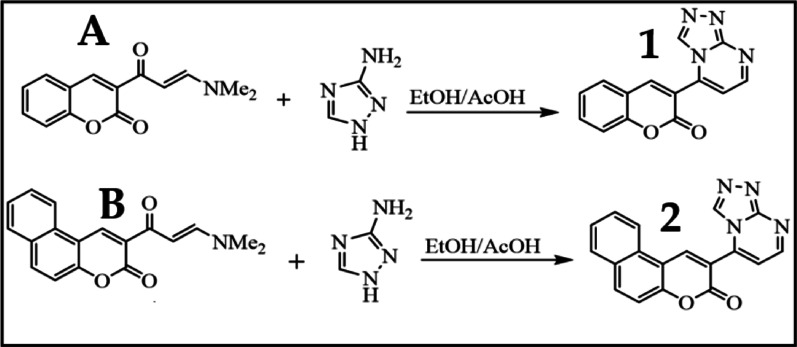
Synthesis of compounds **1**, and **2**

In this method (E)-3-(3-(dimethylamino)acryloyl)-2 H-chromen-2-one was reacted with 1 H-1,2,4-triazol-3-amine to afford compound 1,2,4-triazolopyrimidine derivative **1** as shown in Scheme 2. Similarly, compound (E)-2-(3-(dimethylamino)acryloyl)-3 H-benzo[f]chromen-3-one reacted with 1 H-1,2,4-triazol-3-amine to yield compound **2** as shown in Scheme 2.

To compare the conventional heating conditions and unconventional conditions, compounds **1**, and **2** were synthesised using conventional heating for 6 h following literature preparation by El-Taweel & Elnagdi [60], and Al-Zaydi prior publication [27]. The conventional heating takes 300 min for compound 1, and 240 min for compound **2**, which is considered a long time. The unconventional methods for preparing compound **2** exhibit shorter reaction times (4–20 min) with higher yields (90–100%) than the conventional method, while the best yield and shortest reaction time by using Q-Tube is 100% in 4 min for compound **1**. It should be noted that the expected signals in mass spectrometry (MS), IR, ^1^H-, and ^13^C NMR spectra of compounds **1** and **2** were obtained (Figures S1–S6 of the Supplementary Materials). A comparison between conventional heating and unconventional heating for products **1** and **2** in terms of time, yield, and heating methods is shown in Figure S7, Table S3 of the Supplementary Materials.

### Optical properties

**Fig. 1:**
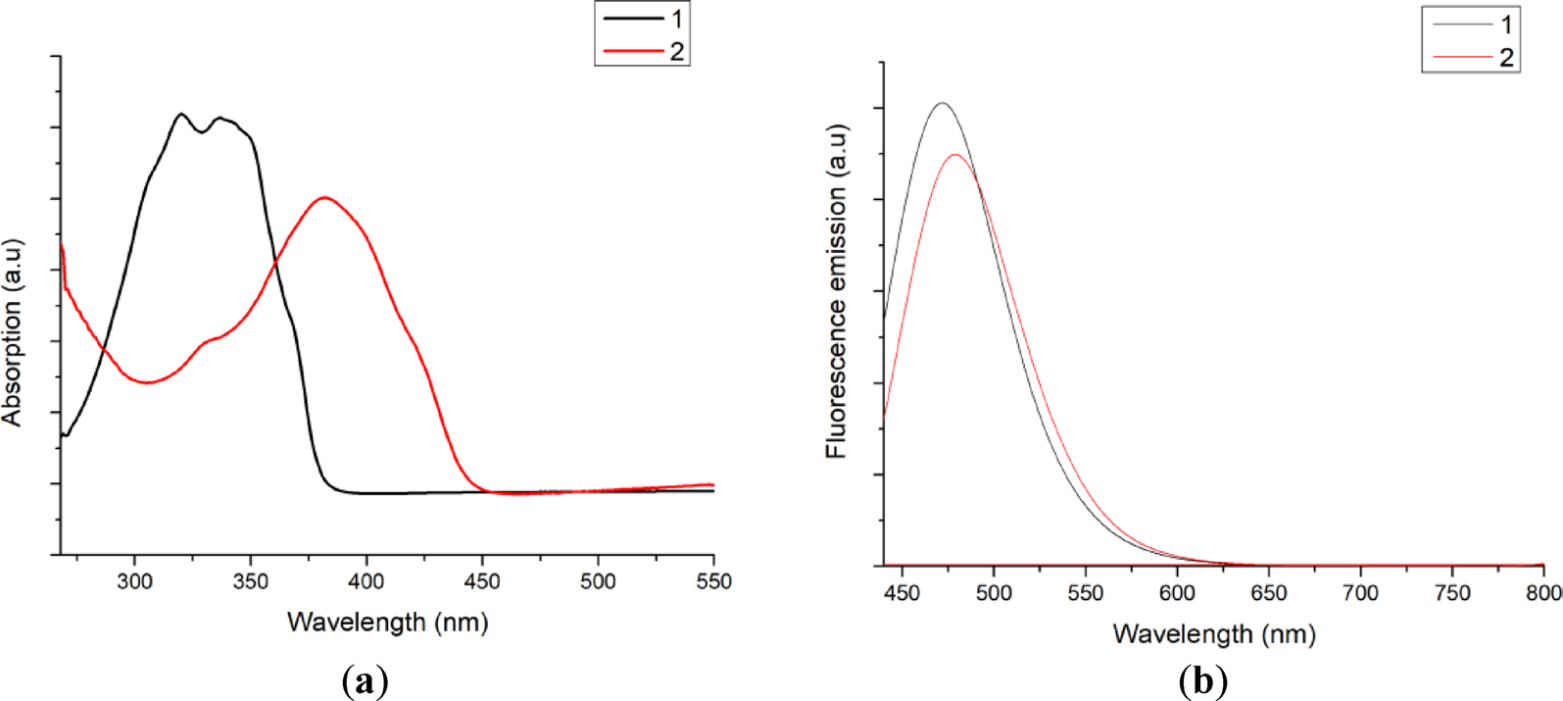
**a** UV-vis absorption spectra of compound **1** (black lines), and 2 (red lines) in DMF solution.** b** Fluorescence emission spectra of compound **1** (black lines), and 2 (red lines) in DMF solution

The absorption and fluorescence spectra of compounds **1** and **2** were measured in dimethyl formamide (DMF) solution. As shown in Fig. 1a, the absorption spectra of **1** and **2** in DMF show absorption bands at 377 nm and 382 nm for **1** and **2**, respectively.

The π-system of the coumarin derivatives has sp^2^ hybridisation, so the spectrum may exhibit an n→π* electronic transition in addition to the π→π* transitions. In both compounds, the coumarin ring acts as a donor while the benzotriazole moiety acts as an acceptor. So, benzo triazole coumarins **2** is a recognised strong intramolecular charge-transfer chromophore system. Therefore, compound **2** displayed a bathochromic shift in comparison to compound **1**.

Figure 1 b shows that both compounds are fluorescent in solution. The presence of additional benzene substituents in 2 may affect their fluorescence spectra by enhancing the π conjugation from the donor to the acceptor, resulting in a red shift with broad emission peaks at 419 nm for compound **2** compared to 414 nm for **1**. The optical properties of compounds **1** and **2** are within the range of other coumarin compounds.

### Docking study

#### Ligand and protein preparation and molecular docking

After downloading the targeted proteins from PDB (ID: 4XUF, 6M2N and 5N2S), they prepared and minimised them by using Schrödinger’s Protein Preparation Wizard. The synthetic compounds and co-crystallised ligands were ready for docking, where energy-minimised 3D structures were generated. The binding pockets within proteins were identified by using a receptor-grid-generation tool to create a glide box around the native co-crystallised ligand. To validate the accuracy of molecular docking, the native ligands were re-docked and compared with the crystallographic ligand. The acceptable root mean square deviation (RMSD) value for superimposition ranges between 0 and 2. The predicted RMSD values for co-crystalised ligands (PDB ID: 4XUF, 6M2N and 5N2S, respectively) were 0.6892, 0.2028, and 0.9573, respectively (Figures S8, 2 and 3). This reflects the similarity between the predicted and actual protein-ligand interactions.

After docking checking, the 3D structures of synthetic compounds were docked utilising extra precision (XP) mode. For each protein, different docking scores were created, including gscore (best for ranking different compounds), emodel (best for ranking conformers), and XP gscore. Table 1 shows the docking scores of synthetic compounds and reference ligands of different proteins. Based on the glide gscores that docked compounds according to their poses, compound **2** showed the highest fitting (-10.909 kcal/mol) to the active site of receptor tyrosine kinase FLT3 (PDB: 4XUF) compared to the native ligand AC220 (-10.688 kcal/mol) that followed by compound **1** (-8.35 kcal/mol) (Table 1). SARS-COV-2 (3CLpro) (PDB: 6M2N) showed a high docking score with the native ligand (Baicalein) (-7.25 kcal/mol), followed by compound **2** (-6.56 kcal/mol) and then compound **1** (-5.586 kcal/mol) (Table 2). Adenosine A1 receptor (PDB: 5N2S) binding centre exhibits high affinity with native ligand (PSB36) (-12.009 kcal/mol) compared to compound **1** (-8.131 kcal/mol) and compound B (-7.331 kcal/mol) (Table 3). Therefore, compound **2** reveals the highest docking score with receptor tyrosine kinase FLT3 (PDB: 4XUF) compared with the native ligand was chosen for further molecular dynamic simulation.

**Table 1:** In Silico Docking results of synthetic compounds (**1**, and **2**) and the co-crystallised ligand against the targeted protein FLT3 (PDB: 4XUF)

Title	Docking score (kcal/mol)	Glide gscore (kcal/mol)	Glide gscore (kcal/mol)	XP GScore (kcal/mol)
Compound 2	− 10.909	− 10.909	− 50.461	− 10.909
Native ligand (AC220)	− 10.624	− 10.688	− 133.843	− 10.688
Compound 1	− 8.35	− 8.35	− 51.063	− 8.35

**Fig. 2:**
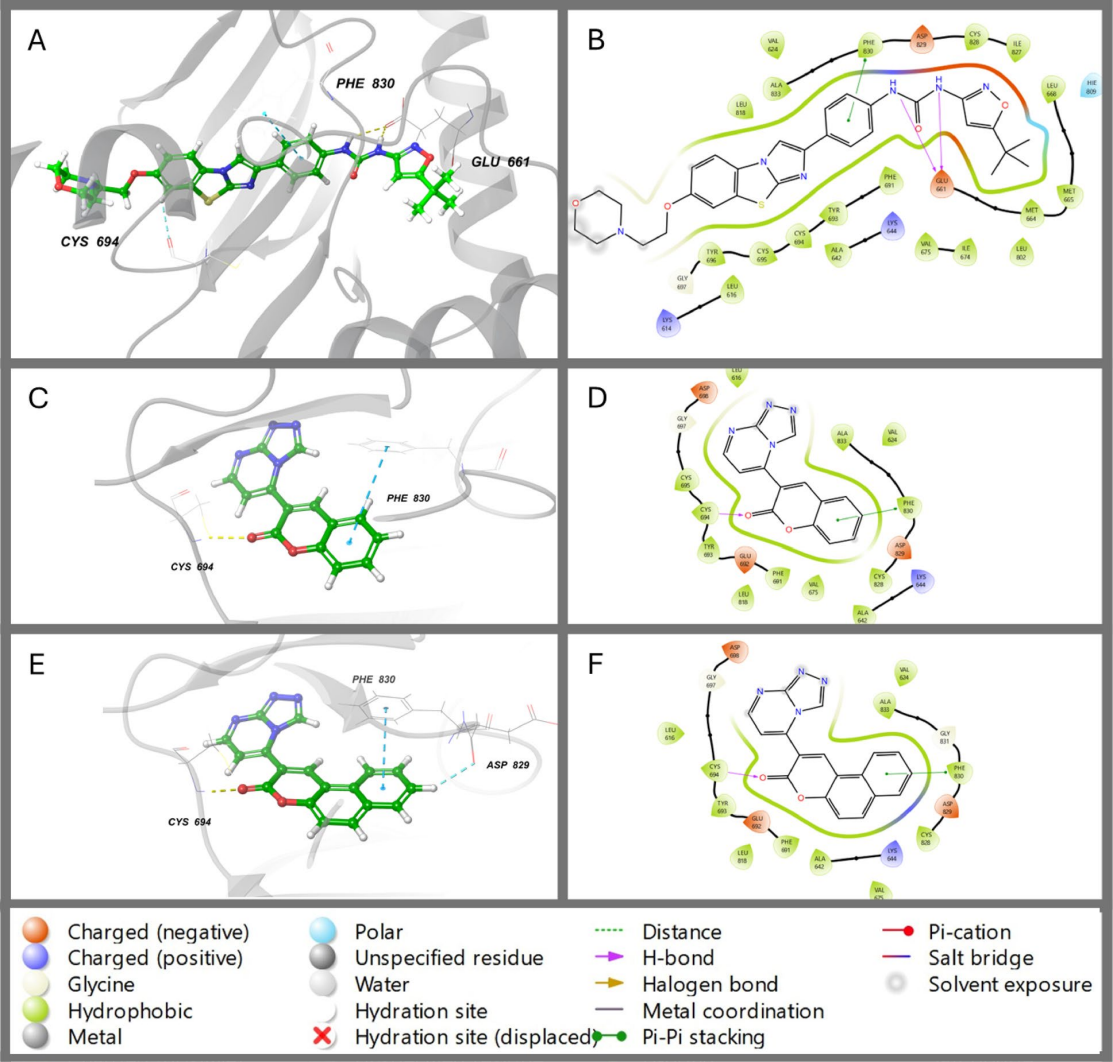
2D and 3D binding modes of co-crystallised ligand (AC220) (**A**, **B**), compound **1** (**C**, **D**) and compound **2** (**E**, **F**) with the active centre of receptor tyrosine kinase FLT3 (PDB ID: 4XUF)

**Fig. 3:**
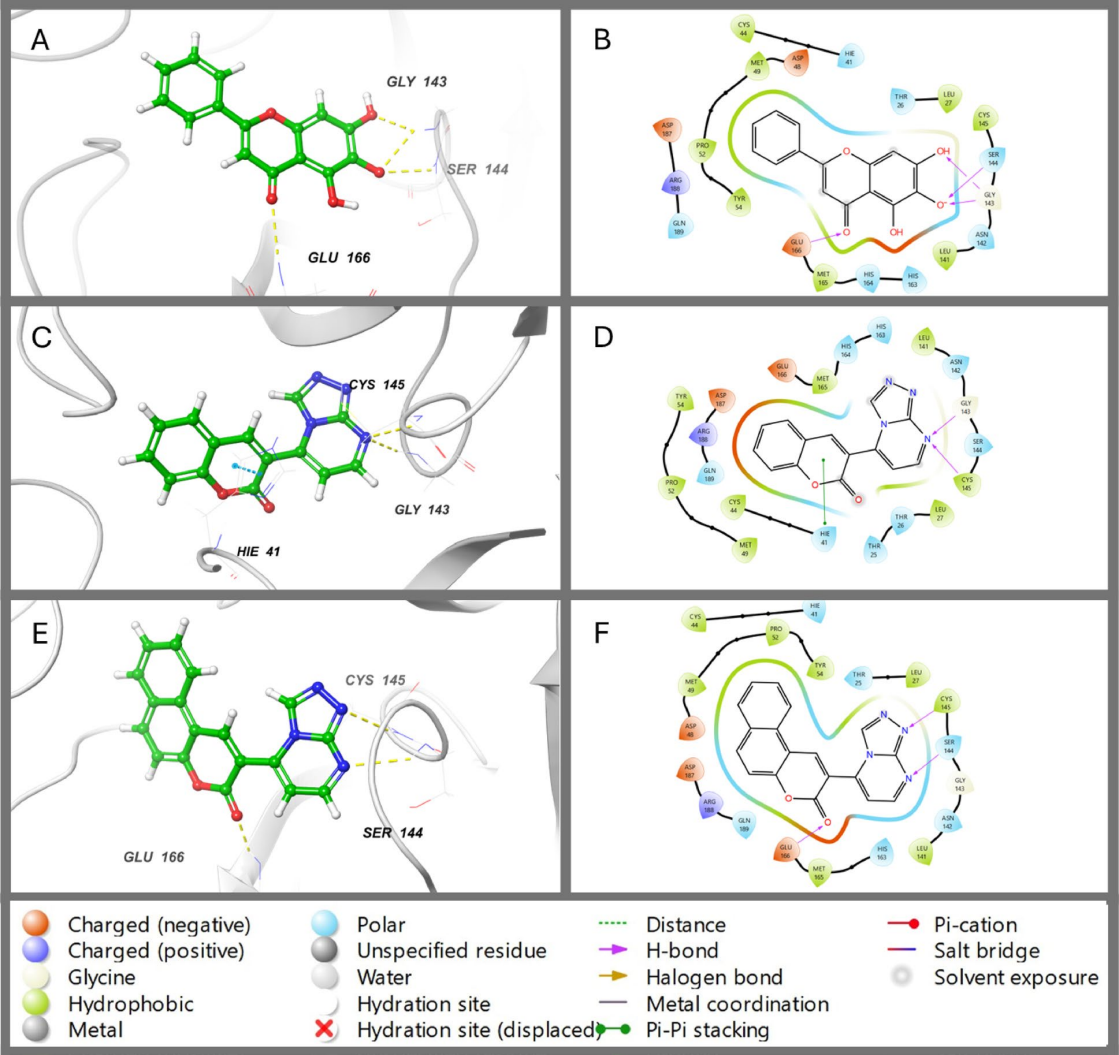
2D and 3D binding modes of co-crystallized ligand (Baicalein) (**A**,** B**), compound **1** (**C**,** D**) and compound **2** (**E**,** F**) with the active center of SARS-COV-2 (3CLpro) (PDB: 6M2N)

The binding interaction of synthetic compounds and co-crystallised ligand with receptor tyrosine kinase FLT3 (PDB: 4XUF) was illustrated in 2D and 3D views (Fig. 2). The CYS-694 residue forms a hydrogen bond with the carbonyl group of the coumarone moiety in both synthetic compounds. The PHE-830 residue engages in pi-pi stacking interactions with the coumarone part of compound **1** and the benzocoumarin moiety of compound **2** (Table 1; Fig. 2C–F). The carbonyl group of the urea in the co-crystallised ligand forms a hydrogen bond with ASP-829, while the amino group of the urea forms two hydrogen bonds with GLU-661 (Fig. 2A and B). Although the native ligand forms three hydrogen bonds (Fig. 3A and B), it did not surpass the docking score of compound **2**, where the ranking of docking score depends on several factors, including bond type, bond number and ligand pose in the binding site.

The 2D and 3D binding modes of ligands and SARS-CoV-2-3CLpro are represented in Fig. 3. The co-crystalised ligand interacts with the protein binding pocket by forming four hydrogen bonds with GLY-143, SER-144 and GLU-166, while compound **2** showed three H-bonds with SER-144, CYS-145 and GLU-166 (Fig. 3A–D). These interactions illustrate the superiority of native ligand and compound **2** docking scores compared with compound **1**, which forms two hydrogen bonds with GLY-143 and CYS-145 and one pi-pi stacking with HIE-41 (Fig. 3E and F; Table 2).

**Table 2:** In Silico Docking results of synthetic compounds (**1**, and** 2**) and the co-crystallized ligand against the targeted protein SARS-COV-2 (3CLpro) (PDB: 6M2N)

Title 1	Docking score (kcal/mol)	Glide gscore (kcal/mol)	Glide gscore (kcal/mol)	XP GScore (kcal/mol)
Native ligand (Baicalein)	− 7.25	− 7.25	− 55.895	− 7.25
Compound 2	− 6.56	− 6.56	− 60.786	− 6.56
Compound 1	− 5.586	− 5.586	− 57.38	− 5.586

**Fig. 4:**
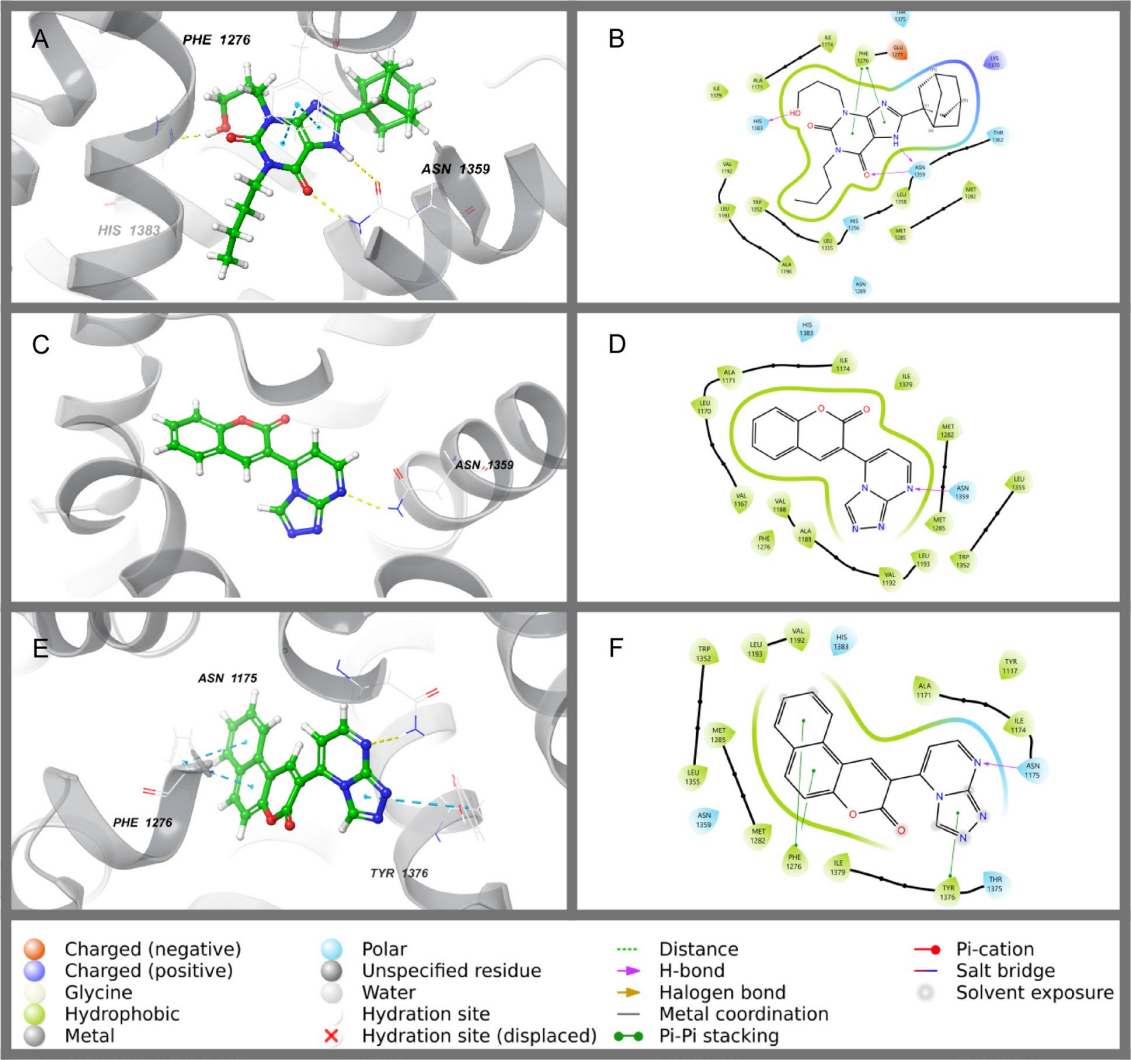
2D and 3D binding modes of co-crystallised ligand (PSB36) (**A**, **B**), compound **1** (**C**, **D**) and compound **2** (**E**, **F**) with the active centres of adenosine A1 receptor A1R (PDB: 5N2S)

**Fig. 5:**
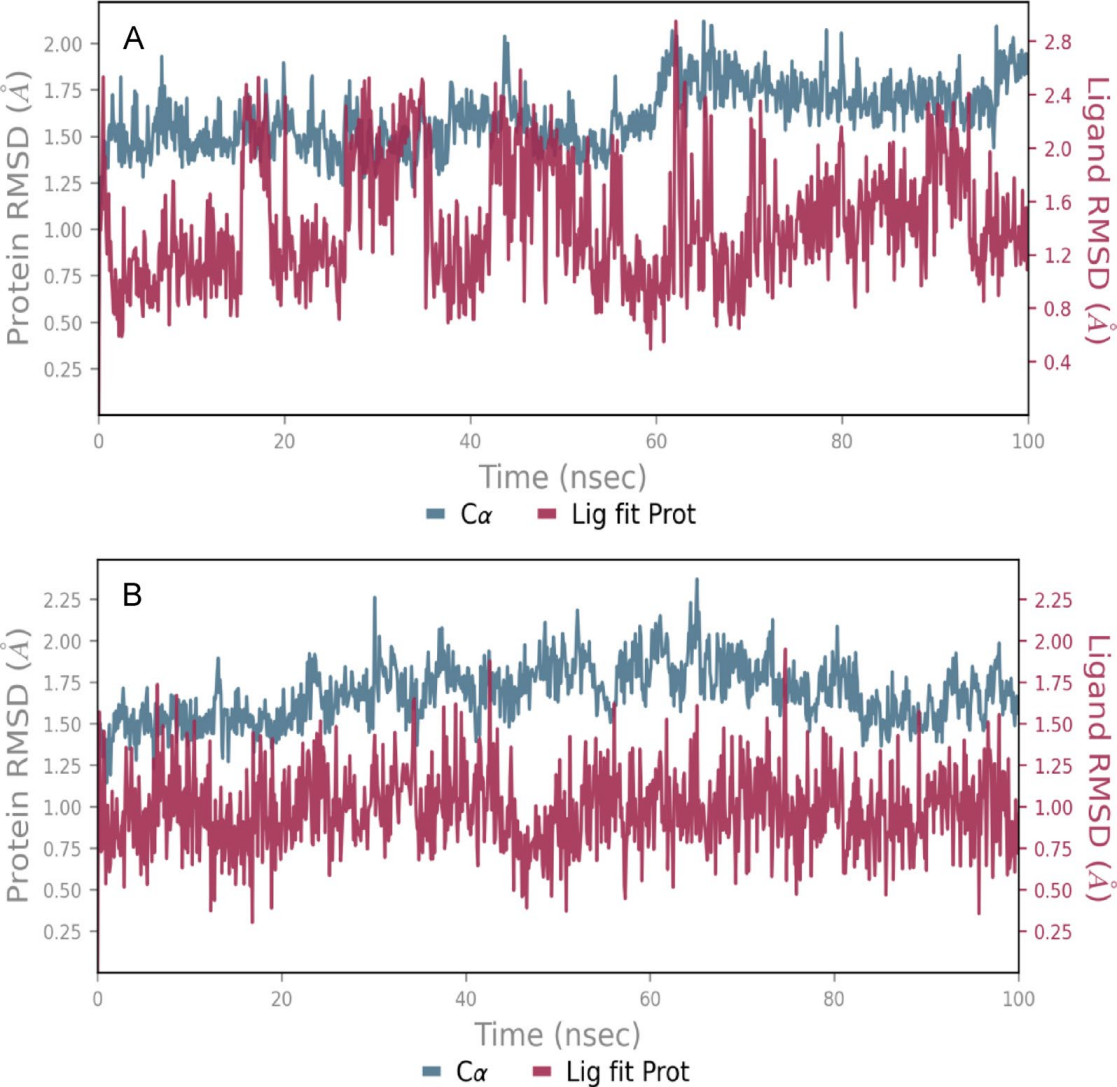
Root mean square deviation (RMSD) plots of the FLT3 protein (PDB ID: 4XUF) in complex with the native ligand AC220 (**A**) and the synthetic compound **2** (**B**)

The protein-ligand interaction of Adenosine A1 receptor with synthetic compounds in 2D and 3D views was illustrated in Fig. 4. The co-crystalized ligand with the top docking score (Table 3) showed three hydrogen bonds with ASN-1359 and HIS-1383, and two pi-pi stacking with PHE-1276, followed by compound **1**, which forms one H-bond with ASN-1359. Although compound **1** interacts with one hydrogen bond, it showed a high docking score compared with compound B, which forms an H-bond with ASN-1175 and three pi-pi stacking with TYR-1376 and PHE-1276, which may be due to the proper fitting with the binding site (Fig. 5E and F). Since synthetic compound **2** exhibited a more favorable docking score than the native ligand AC220, molecular dynamics (MD) simulations were conducted to further investigate the stability and interaction profile of the compound within the active site of FLT3 (PDB: 4XUF).

**Table 3:** In Silico Docking results of synthetic compounds (**1**, and 2) and the co-crystallised ligand against the targeted protein adenosine A1 receptor (PDB: 5N2S)

Title 1	Docking score (kcal/mol)	Glide gscore (kcal/mol)	Glide gscore (kcal/mol)	XP GScore (kcal/mol)
Native ligand (PSB36)	− 12.009	− 12.009	− 79.328	− 12.009
Compound **2**	− 8.131	− 8.131	− 47.034	− 8.131
Compound **1**	− 7.331	− 7.331	− 50.025	− 7.331

### Molecular dynamics (MD) simulations

MD simulations were performed using the Desmond module within the Schrödinger software suite to investigate the dynamic behaviour and binding stability of ligand-protein complexes under near-physiological conditions. The native ligand (AC220) and compound **2** were selected based on docking scores. Root mean square deviation (RMSD) analysis was utilised to monitor the structural stability of the FLT3 protein (PDB ID: 4XUF) and the ligands over the simulation timeline. RMSD plots were generated to reflect deviations of the protein (left Y-axis) and ligand positioning relative to the protein (right Y-axis), as illustrated in Fig. 5. The RMSD trajectory of both FLT3-AC220 complex (Fig. 5A) and FLT3-compound **2** complex (Fig. 5B) showed that the protein remained structurally stable throughout the 100 ns simulation, with slight fluctuations within acceptable limits (1.0–3.0 Å). Notably, Compound **2** demonstrated a more stable and consistent RMSD, averaging around 1.0 Å, suggesting a robust and sustained interaction with the binding site. These findings suggest that both ligands form stable complexes with FLT3 under simulated physiological conditions, with compound **2** potentially offering enhanced binding stability compared to the reference ligand.

**Fig. 6:**
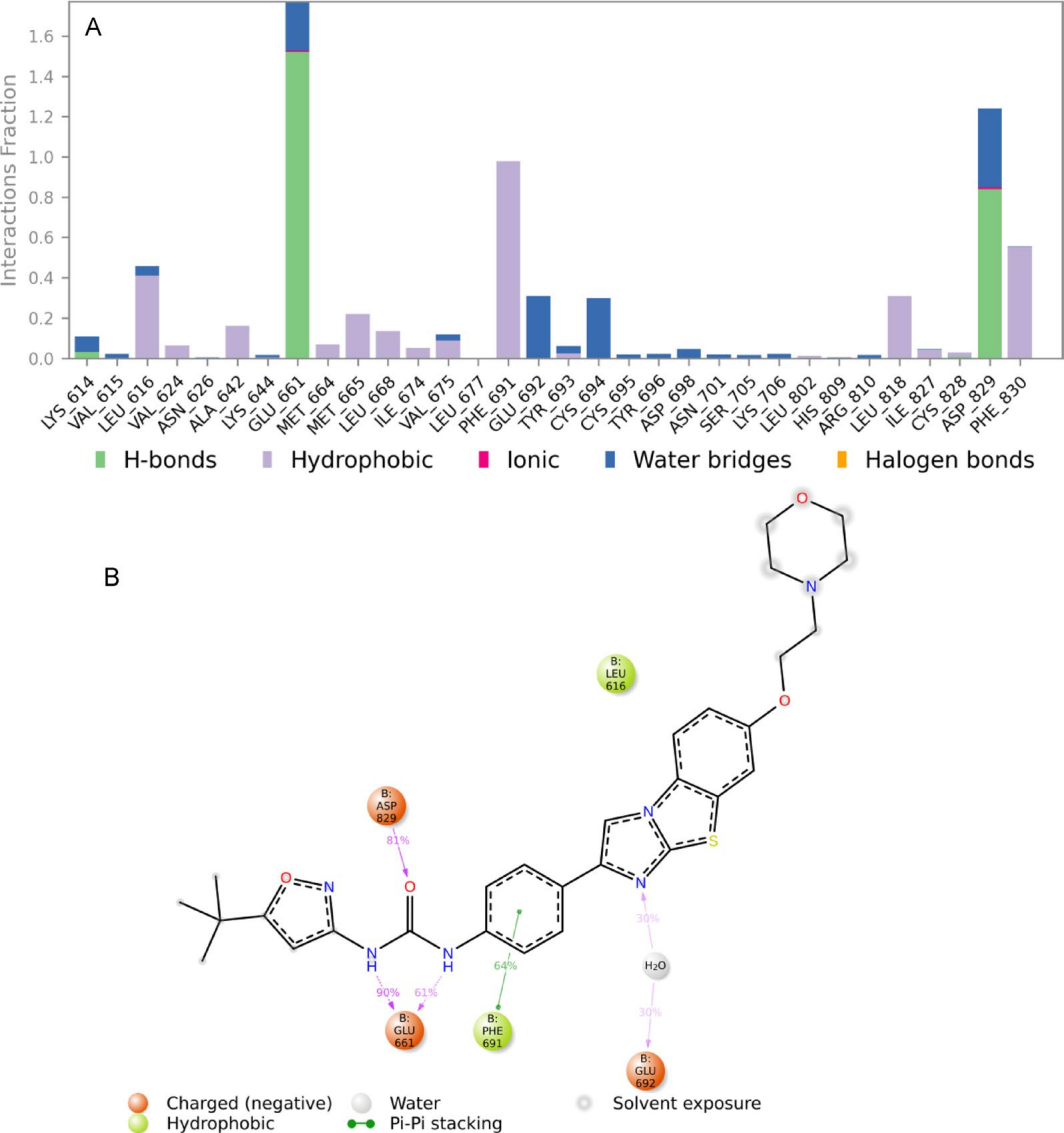
**A** The stacked bar chart represents the interaction between the native ligand AC220 and FLT3 protein (PDB ID: 4XUF) residues. **B** The schematic diagram shows the detailed 2D atomic interactions of AC220 with FLT3 that occurred >30% of the simulation time in the selected trajectory

**Fig. 7:**
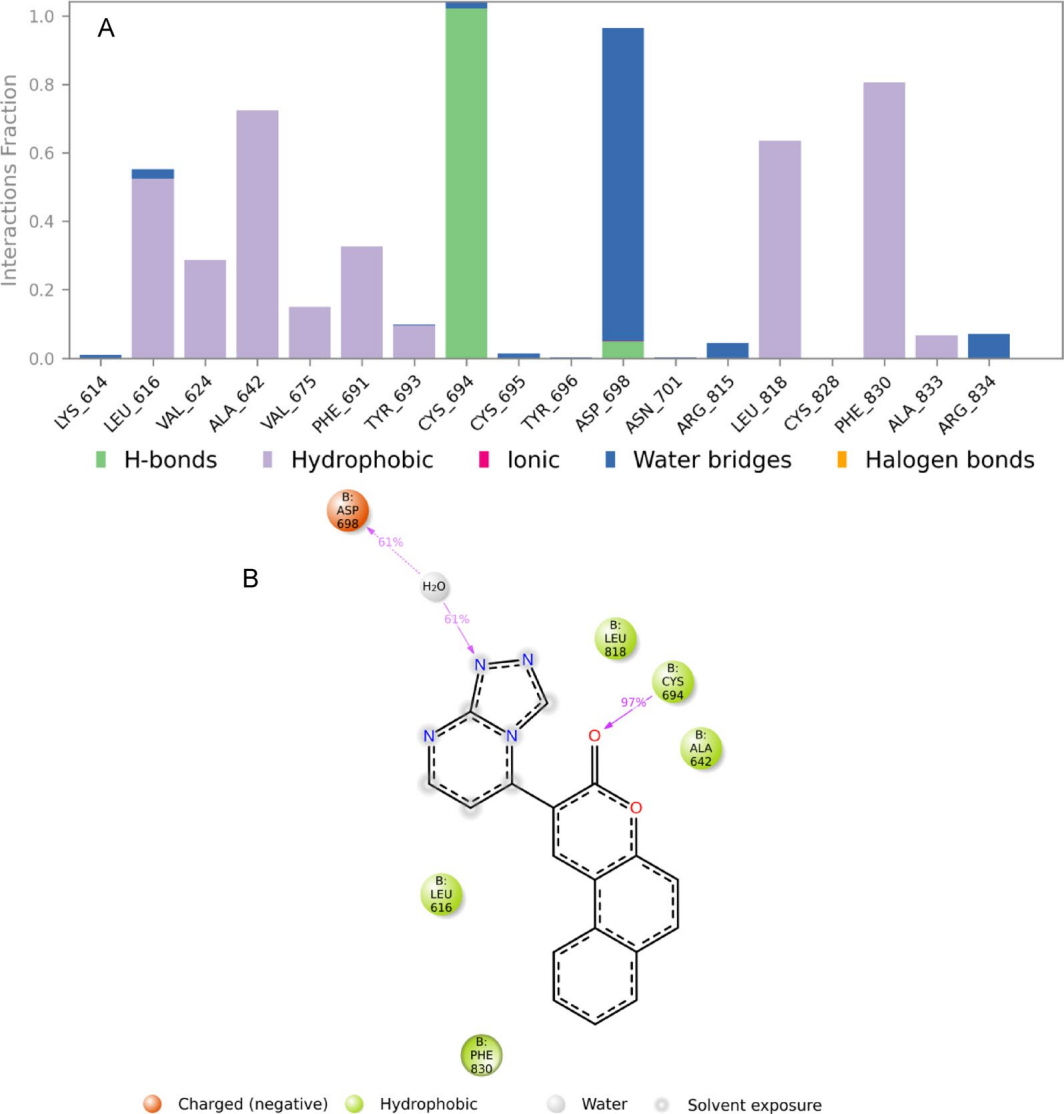
**A** The stacked bar chart represents the interaction between compound **2** and FLT3 protein (PDB ID: 4XUF) residues. **B** The schematic diagram shows the detailed 2D atomic interactions of compound **2** with FLT3 that occurred > 30% of the simulation time in the selected trajectory

Figures 6 and 7 highlight the key amino acid residues within the FLT3 protein binding pocket that interact with the native ligand AC220 and synthetic compound **2**, respectively. The stacked bar charts (Figs. 6A and 7A) quantify the interaction frequency, normalised over the 100 ns simulation trajectory. A bar height of 0.3, for example, indicates that the specific interaction persisted for 30% of the simulation time, while values exceeding 1.0 reflect multiple types of interactions with the same residue. Interaction types are colour-coded: hydrogen bonds (green), hydrophobic contacts (violet) and water bridges (blue).

In Fig. 6A, the interaction profile of AC220 with FLT3 residues reveals that Glu661 and Asp829 form prominent hydrogen bonds, with interaction fractions around 1.5 and 0.9, respectively. Hydrophobic contacts are observed with Leu616 (~ 0.4), Phe691 (~ 1.0), Leu818 (~ 0.3), and Phe830 (~ 0.6). Additional water bridges are observed with Glu692, Cys694, and Asp829, each contributing approximately ~ 0.3 interaction fractions. Figure 6B presents a 2D schematic of the binding interactions between AC220 and FLT3. The dimethylurea moiety of AC220 forms hydrogen bonds with Glu661 (151%) and Asp829 (81%), aligned with the docking predictions. Phe691 engages in hydrophobic interactions with AC220’s benzene ring (~ 64%), and Glu692 forms a water bridge with the ligand’s imidazole ring (~ 30%).

In Fig. 7A, the synthetic compound **2** shows predominantly hydrophobic interactions with FLT3 residues such as Leu616 (~ 0.5), Ala642 (~ 0.7), Leu818 (~ 0.6), and Phe830 (~ 0.8), sustained over more than 30% of the simulation. Notably, Asp698 participates in a water bridge (~ 0.9), while Cys694 establishes a stable hydrogen bond (~ 1.0). Figure 7B illustrates the 2D interaction map of compound **2** within the FLT3 binding site. The carbonyl group of the benzocoumarin scaffold forms a hydrogen bond with Cys694 (97%), consistent with the docking results. Furthermore, a water bridge is formed between the triazole moiety of the ligand and Asp698, occurring for 61% of the simulation duration.

### In silico ADME predictions, and biological study

The coumarin pharmacophore has been regarded as the best small-molecule scaffold for the development of novel medications due to its drug-like characteristics and, more importantly, its relationship with numerous pharmacological actions. The coumarin pharmacophore is found in various clinically used medication candidates, including some well-known antibiotics. Both in silico protocol and computational molecular docking studies have become as valuable as modelling tools for drug design. In silico structure-based protocols are generally utilised to interpret the molecular aspects of ligand-protein interactions during drug discovery against various targets. This approach becomes essential, especially with fatal and emerging diseases [61, 62] such as Zika, Ebola, the Middle East respiratory syndrome (MERS) coronavirus, SARS coronavirus, and coronavirus 2 (SARS-CoV-2) [63, 64].

However, compounds with the strongest binding interactions with the receptor might not always be the most effective medications. They typically qualify as potential medications when their pharmacodynamic and pharmacokinetic qualities are sufficient. Therefore, the development of prospective drugs may benefit from the ADMET profile (absorption, distribution, metabolism, excretion, and toxicity). This method efficiently, clearly, and accurately predicts in *vivo* performance, which is important for the investigation of a potential drug. Therefore, the pharmacokinetic predictions of **1** and **2** were calculated. From the data provided in Table 4, it is evident that all the chosen compounds comply with the drug likeness guidelines. There is a general guideline for assessing the potential of a chemical compound to be an orally active drug for humans based on its chemical and physical properties, such as Lipinski (Pfizer) [65], Veber (GSK) [66], Ghose [67], Egan [68], and Muegge [69] rules.

Compounds (**1** and **2**) satisfy Lipinski (Pfizer), Veber (GSK), Ghose, Egan, and Muegge rules criteria as shown in Table 4, making them potential drug-like molecules. Furthermore, compounds (**1** and **2**) displayed great bioavailability, indicating that the prospective medication is well absorbed and distributed.

**Table 4:** Predicted Lipinski (Pfizer), Veber (GSK ), Ghose, Egan, Muegge rules, and medicinal chemistry properties of compounds **1** and **2**

Compound ID	Lipinski (Pfizer) rules ^a^	Veber (GSK) rules	Ghose rules	Egan Rules^d^	Muegge Rules^e^	Medicinal Chemistry
	Drug-likeness	Drug-likeness	Drug-likeness	Drug-likeness	Drug-likeness	Synthetic accessibility
1	Yes	Yes	Yes	Yes	Yes	0.55
2	Yes	Yes	Yes	Yes	Yes	0.55

^a^Lipinski (Pfizer) rules [65]: (Mw ≤ 500, MlOGP ≤ 4.15, N or O ≤ 10, NH or OH ≤ 5)

^b^Veber (GSK) rules [66]: (Rotable bonds ≤ 10, TPSA ≤ 140)

^c^Ghose [67]: (160 ≤ Mw ≤ 480, -0.4 ≤ WLOGP ≤ 5.6, 40 ≤ MR ≤ 130, 20 ≤ atoms ≤ 70)

^d^Egan [68]: (WLOGP ≤ 5.88, TSPA ≤ 131.6)

^e^Muegge [69]: (200 ≤ Mw ≤ 600, -2 ≤ XLOGP ≤ 5, TPSA ≤ 150, #ring ≤ 7, #C > 4, # hetroatoms > 1, Rotable bonds ≤ 15, H-bond acc. ≤10, H-bond don. ≤5)

Additionally, the chosen compounds were evaluated for their synthetic accessibility on a scale of 1 to 10, where 1 represents easy synthesis and 10 represents complex synthesis. The synthetic accessibility scores for compounds **1** and **2** are 2.76 and 2.97, respectively, indicating that they are relatively easy to synthesise. Another significant metric for estimating drug transport qualities is the topical polar surface (TPSA), which represents passive molecule transport across cell membranes. This realistic polar surface area metric eliminates the need to compute the ligand’s 3D structure or pick the requisite biological conformation or conformations by utilising functional group contributions from a large library of structures [70]. A TPSA score above 140 Å² often indicates insufficient penetration across the cell membrane. Compounds with TPSA less than 70 Å² have a higher ability to pass through cell membranes than those with TPSA between 100 and 130 Å². Compounds **1**, **2** display TPSAs of 73.29Å². Both compounds were anticipated to be skin permeable since their skin permeability constants were less than − 2.5 log Kp, indicating poor permeability (-6.36 cm/s for **1**, and − 5.78 cm/s for **2**). Compound **1** displayed excellent water solubility (-logS value of -3.49), while compound **2** (-logS value of -4.59) shows moderate water solubility, and thus, much fewer problems may be encountered during drug formulation.

**Fig. 8:**
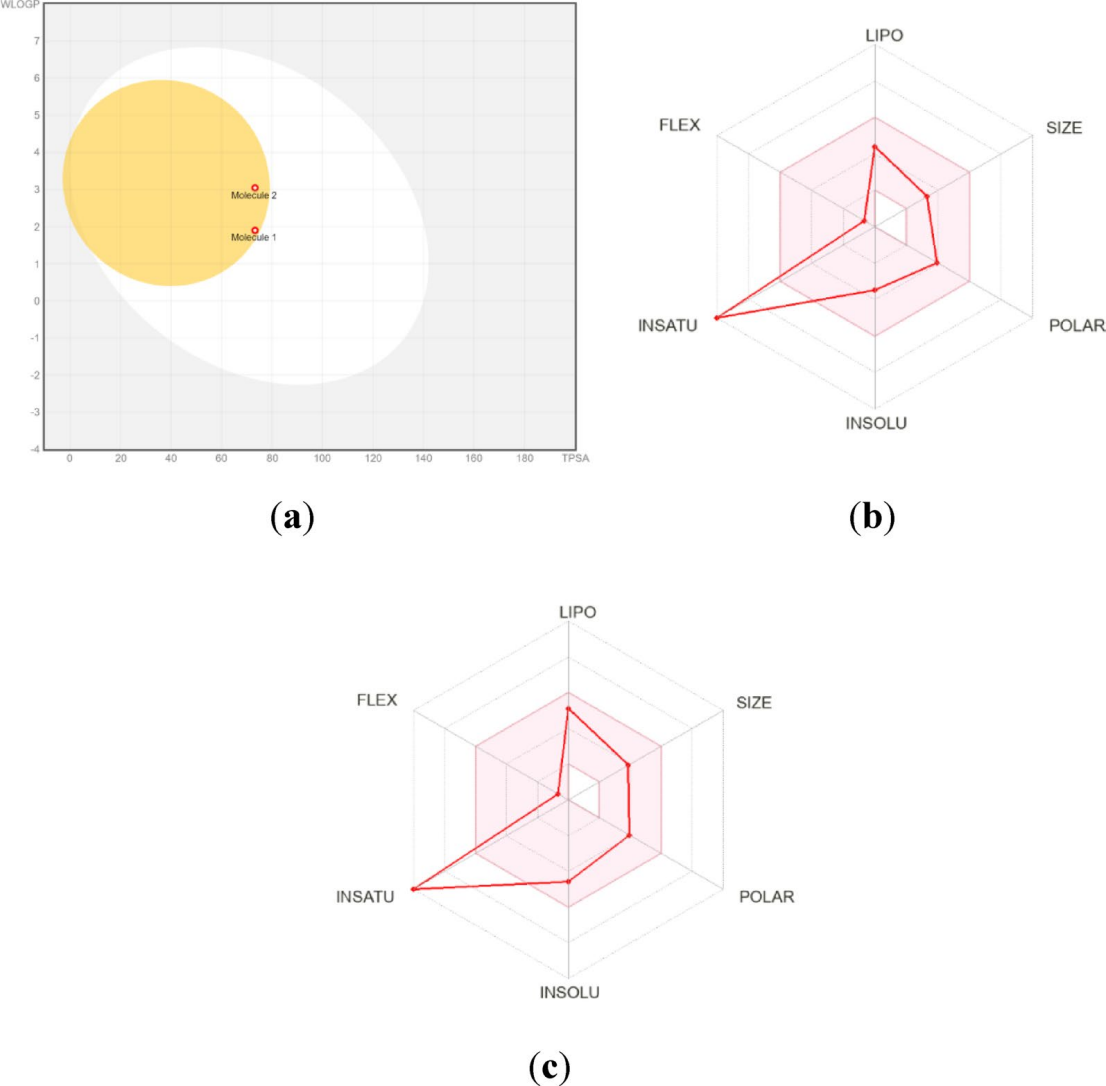
**a** Graph of the dependence of lipophilicity on the polarity of the studied molecules **1** and **2** determined by the BOILED-Egg method [54, 73]. Bioavailability radar [75] graph of derivatives **1** (**b**) and **2** (**c**) obtained from Swiss ADME prediction [54]. The pink area represents the allowed values of drug likeness properties of the molecule

All synthesised compounds have a displayed lipophilicity (log P) of < 5, indicating good permeability to the target tissue. Additionally, the relationship between the lipophilicity and polarity of the examined compounds **1** and **2** was also investigated utilising the BOILED-Egg method (Brain or Intestinal Estimated permeation) of evaluating penetration via the brain or intestines as an accurate predictive model [71–73]. This analysis is illustrated in Fig. 8a. The investigated substances exhibit good permeability through the blood-brain barrier (BBB) (yellow area) and proper binding to human blood albumin (HIA) (white area). Figure 8 also indicates that both compounds **1** and **2** can not become p-glycoprotein substrates with negative consequences (red points). Both compounds were predicted to be highly absorbed from the gastrointestinal tract (GIT) and enter the BBB efficiently. The PGP- represented by red dots is for molecules calculated not to be actively collapsed from the CNS by the P-glycoprotein. The predicted oral toxicity of compounds **1** and **2** was LD50 350 mg/kg, putting both of them in the class IV toxicity class (300 < LD_50_ ≤ 2000).

The Bioavailability Radar is shown for a quick assessment of drug-like properties. The Bioavailability Radar analyses six physicochemical characteristics, including lipophilicity, size, polarity, solubility, flexibility, and saturation. For the molecule to be a drug-like chemical, the Bioavailability Radar graph must be confined inside a pink zone. If the graph is in the pink area, it can be classified as a drug-like compound for the chemical. Figure 8 (**a** and **b**) displays bioavailability radar plots created using the SwissADME web application. For both compounds, only the instauration characteristics are out of the desired range, while all the others fit into the red-depicted area, accounting for an assumed moderate oral bioavailability.

It should be noted that drug companies suffer enormous financial losses as a result of post-marketing drug failure caused by ADME and toxicity [74]. ADME studies are critical parameters to investigate during the drug discovery process in order to limit the risk of pharmacokinetics-related clinical failure. Therefore, developing novel chemicals with therapeutic applications, ADME, and toxicity investigations should not be ignored. All of our compounds were examined for ADME and toxicity prediction [74]. Both compounds showed great Physicochemical, Pharmacokinetics, Drug-likeness, and Medicinal chemistry properties, with potential targeting to four deadly diseases. The main issue with those compounds is the permeability through the blood-brain barrier (BBB), which might need more investigation. The recent discovery may pave the way for the production of improved triazolo pyrimidine coumarin and benzocoumarin via chemical modification to improve the ADME properties of the compounds, resulting in future developments in leukaemia, SARS-CoV-2, heart failure and Alzheimer’s disease.

## Conclusions

Compounds **1** and **2** were synthesised using green methodology by using Q-Tube to afford compounds in higher yields and shorter times. In Silico evaluation of both compounds as potential early-stage small molecule inhibitors of three targets, which cause four life-threatening diseases: leukaemia FLT3 (PDB: 4XUF), SARS-COV-2 (3CLpro) (PDB: 6M2N), and adenosine A1 receptors A1R (PDB: 5N2S), which could cause heart failure and Alzheimer’s disease. It was discovered that both compounds show promising results against the three targets. The results of this study showed the effectiveness of these two synthetic coumarin triazolo pyrimidine derivatives in modulating several illnesses according to an in silico approach. These novel synthetic compounds exhibited anticancer activity against acute myeloid leukaemia AML by targeting receptor tyrosine kinase FLT3 and antiviral activity against Coved 19 by targeting SARS-CoV-2 3CL protease (3CL pro). Moreover, it can modulate many cardiovascular illnesses like heart failure and neurodegenerative disorders such as Alzheimer’s by targeting the adenosine A1 receptor. Moreover, compounds (**1** and **2**) satisfy Lipinski (Pfizer), Veber (GSK)rule, Ghose rules, Egan Rules, and Muegge rules, making them potential drug-like molecules, with good ADMET properties.

## Supplementary Information

Below is the link to the electronic supplementary material.

Supplementary Material 1.

## Data Availability

The data presented in this study are available in this article and the supplementary Materials.
