# Supplementary material for: Triazolo pyrimidine derivatives of coumarin and benzocoumarin: green synthesis, biological activity screening study with in silico evaluation
Source: BMC Chem. 2025 Oct 14;19(1):276. doi: 10.1186/s13065-025-01636-6 (PMC12519810; doi:10.1186/s13065-025-01636-6)
Supplement: Supplementary file 1 — Supplementary Material 1. [file 13065_2025_1636_MOESM1_ESM.docx]

**Supporting Information:**

Triazolo Pyrimidine Derivatives of Coumarin and Benzocoumarin: Green Synthesis, Biological Activity Screening Study with in Silico Evaluation.

**Hawazen M. Hassanain 1,*, Meaad J. Al-Zahrani 1, Roaa Mahdi Alreemi,2 Huda A. Al-Ghamdi 1, Ahlam I. Al-Sulami 1 and Khadijah M. Al-Zaydi 1,***

102

00

98

96

94

92

90

88

86

84

82

80

78

76

74

3060

902.58cm-1

540.68cm-1

456.10cm-1

470.17cm-1

631.85cm-1

573.47cm-1

1488.96cm-1

1603.51cm-1

1296.33cm-1

1453.09cm-1

1560.77cm-1

999.73cm-1

968.27cm-1

1072.01cm-1

1212.17cm-1 855.16cm-1

1577.39cm-1

1325.52cm-1

1352.99cm-1

922.59cm-1

1644.42cm-1

1781.52cm-1

879.88cm-1

1191.61cm-1

1153.31cm-1

1088.50cm-1

1175.49cm-1

1745.27cm-1

697.52cm-1

755.88cm-1

1

4000

3500 3000 2500 2000 1500 1000 500 400

cm-1

%T

**Fig.S1** IR. of compound 1.

102

00

98

96

94

92

90

88

86

84

82

80

78

76

74

3060

902.58cm-1

540.68cm-1

456.10cm-1

470.17cm-1

631.85cm-1

573.47cm-1

1488.96cm-1

1603.51cm-1

1296.33cm-1

999.73cm-1

968.27cm-1

1453.09cm-1 1072.01cm-1

1560.77cm-1

1212.17cm-1

1577.39cm-1 1325.52cm-1

1352.99cm-1

1644.42cm-1

855.16cm-1

922.59cm-1

879.88cm-1

1781.52cm-1

1191.61cm-1

1153.31cm-1

1088.50cm-1

1175.49cm-1

1745.27cm-1

697.52cm-1

755.88cm-1

1

4000

3500 3000 2500 2000 1500 1000 500 400

cm-1

%T

**Fig.S2** IR. of compound 2.


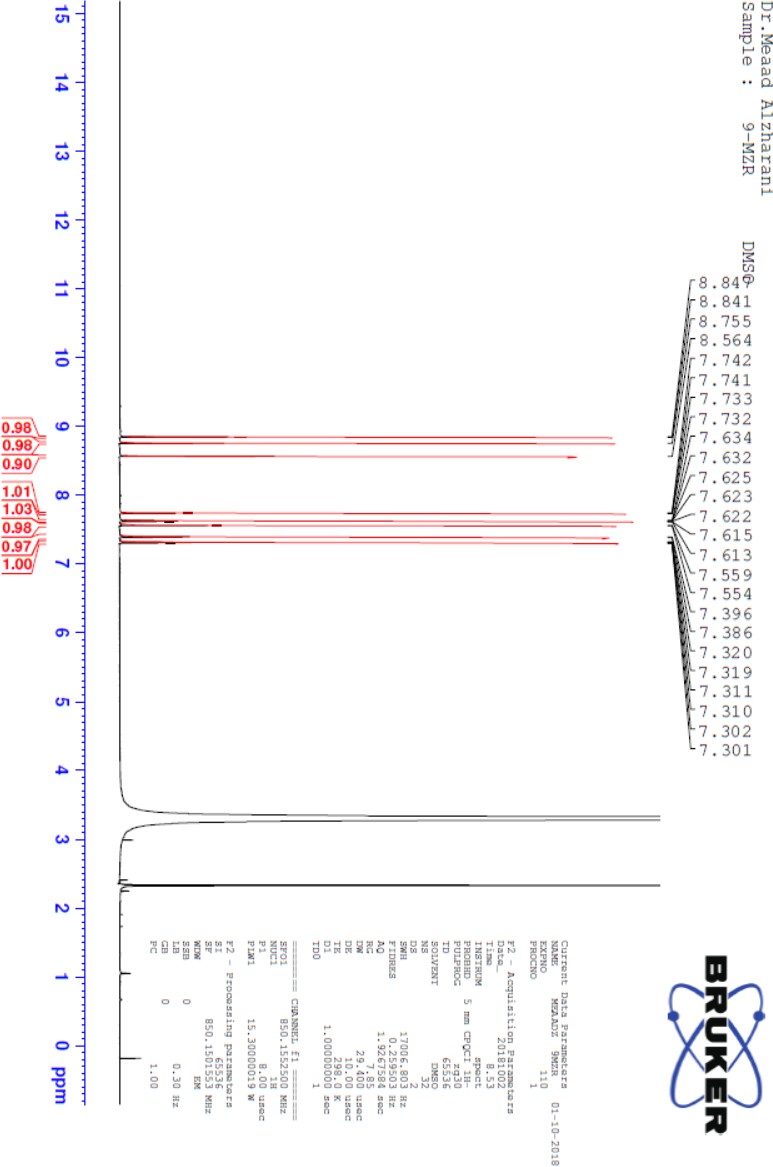


**Fig.S3** ^1^H-NMR. of compound 1


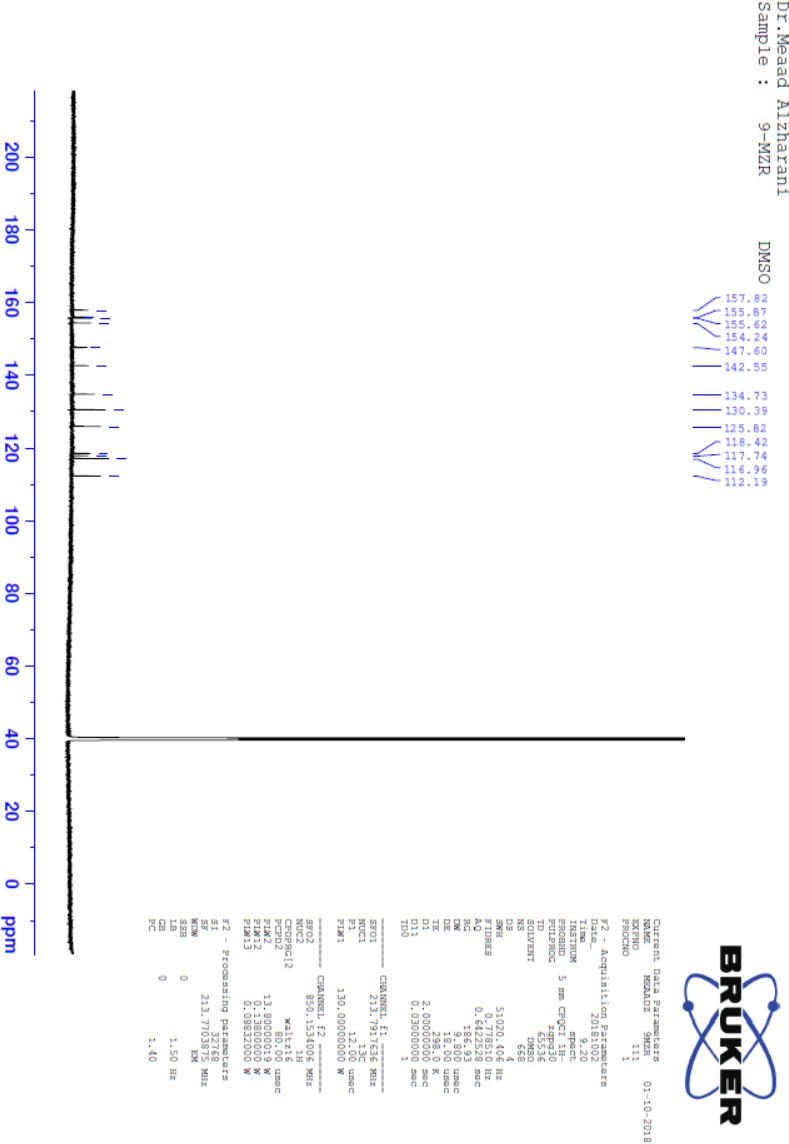


.

**Fig. S4** ^13^C-NMR. of compound 1.


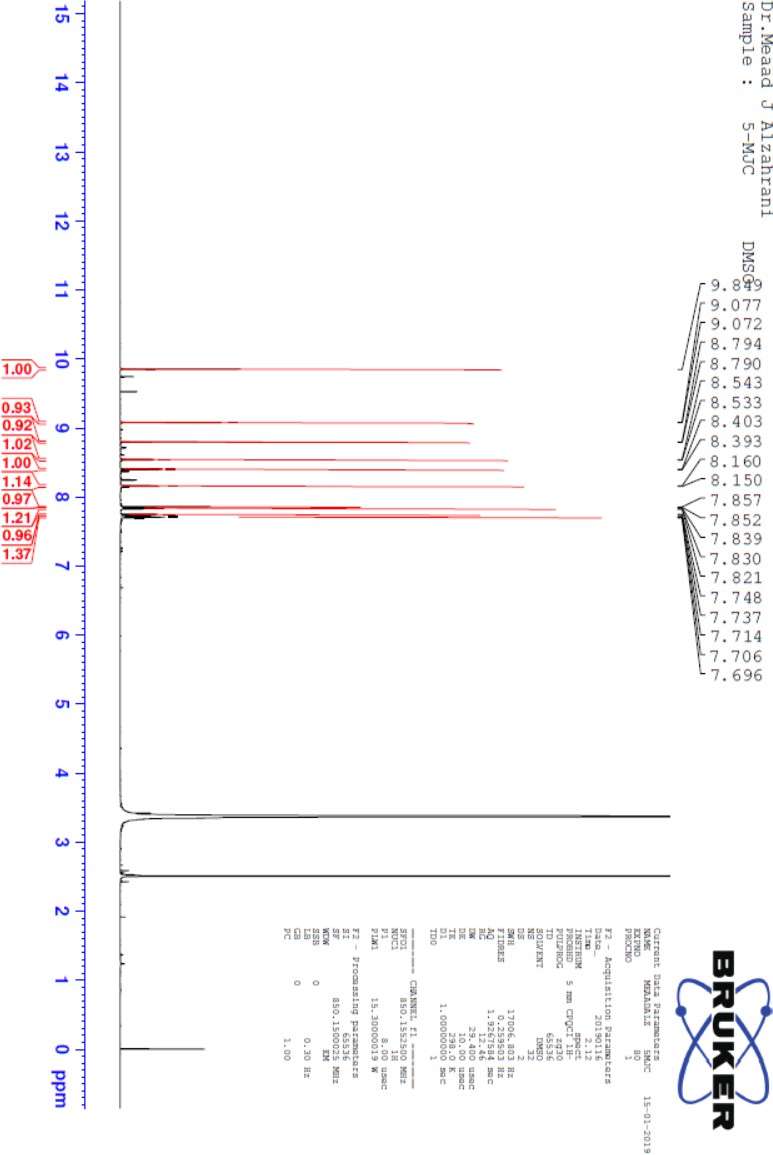


**Fig.S5** ^1^H-NMR. of compound 2.


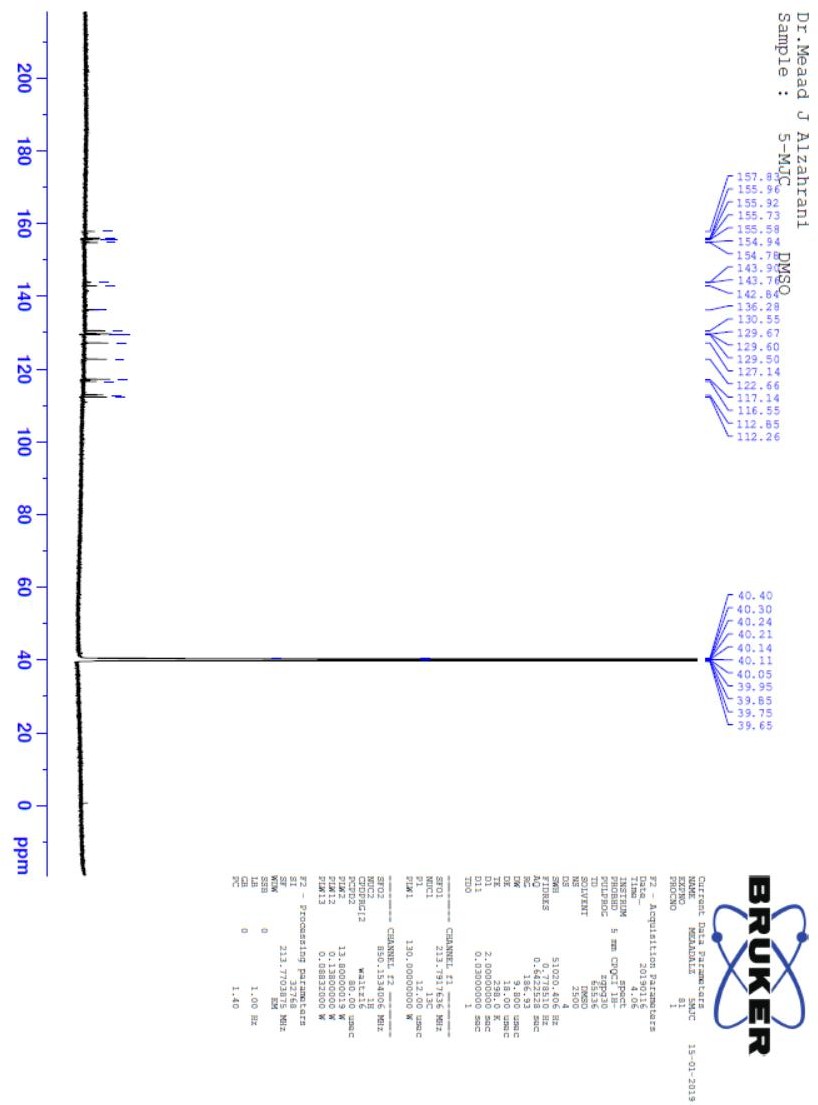


**Fig. S6** ^13^C-NMR. of compound 2.

**Table.S3** Comparison between conventional heating, and unconventional for products 1,

.and 2

|  | Δ |  | Q-Tube |  | US |  |
| --- | --- | --- | --- | --- | --- | --- |
| Product | Y% | Time (min) | Y% | Time (min) | Y% | Time(min) |
| **1** | 66 | 300 | 80 | 5 | 99 | 300 |
| **2** | 80 | 240 | 100 | 4 | 96 | 20 |

Δ Q - T U B E US

T I M E

Y%

T I M E

Y%

T I M E

Y%

100

50

0

Compound 1

Compound 2

350

300

250

200

150

**Fig. S7** Time and yield of compounds 1, and 2 by conventional heating, and

.unconventional heating method


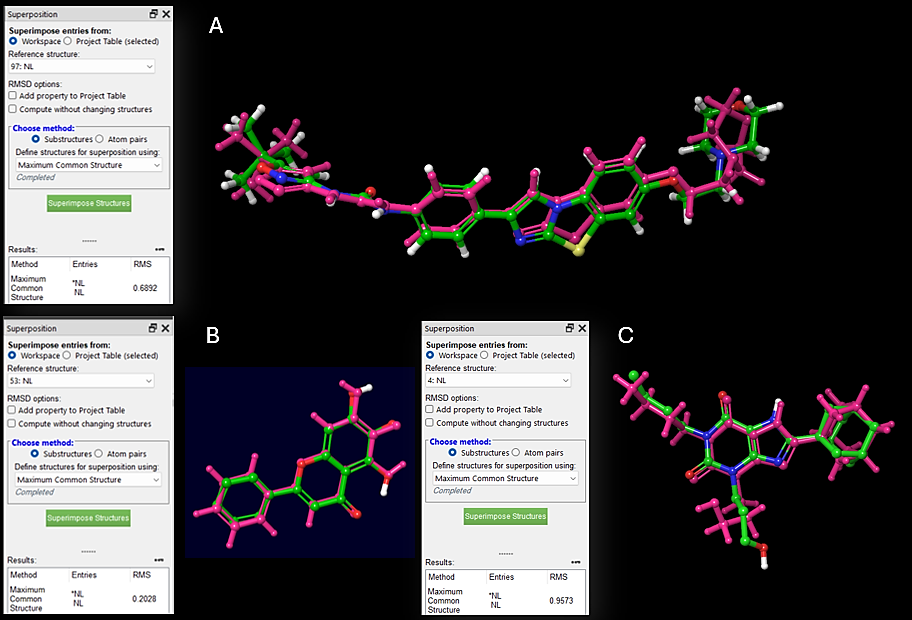


**Fig. S8.** 3D structure of re-docked (green) aligned on co-crystallized (pink) ligand of PDB ID: 4XUF (A), 6M2N (B) and 5N2S (C).
